# PHYSICAL FUNCTION, DEPRESSION, AND COGNITIVE FUNCTION OF OLDER CHINESE AMERICANS: CROSS-LAGGED MODEL

**DOI:** 10.1093/geroni/igad104.3527

**Published:** 2023-12-21

**Authors:** Soonhyung Kwon, Yuyang Zhu, Fengyan Tang, Yanping Jiang

**Affiliations:** Rutgers, The State University of New Jersey, New Brunswick, New Jersey, United States; Rutgers University, Piscataway, New Jersey, United States; University of Pittsburgh, Pittsburgh, Pennsylvania, United States; Rutgers University, New Brunswick, New Jersey, United States

## Abstract

**Introduction:**

Understanding the complex interactions of physical function, depression, and cognitive function is crucial for understanding healthy aging. However, few studies have empirically tested these relationships. This study examined the directional relationships of physical function, depression, and cognitive function among older Chinese Americans.

**Methods:**

This study included a subsample of participants (n=2,811) from the Population Study of Chinese Elderly in Chicago who completed the baseline survey during 2011-2013 (W1) and at least one of the three follow-up surveys conducted in 2013-2015 (W2), 2015- 2017 (W3), and 2017-2019 (W4). Physical function, depression, and cognitive function were measured by a battery of physical mobility, the 9-item Patient Health Questionnaire, and cognitive performance tests (executive function, memory, and cognitive impairments). A random intercept cross-lagged panel model was performed to examine the bidirectional relationships among physical function, depression, and cognitive function.

**Results:**

The cross-lagged panel model showed that higher levels of depression at W1 were associated with poorer physical function at W2. In turn, poorer physical function at W2 was related to higher levels of depression at W3. Higher levels of depression (W2) were linked to poorer physical function at W3. Poorer physical function at W2 was associated with poorer cognitive function at W3. Finally, poorer cognitive function at W3 was associated with poorer physical function and higher levels of depression at W4.

**Discussion:**

Our results support a bidirectional relationship between physical function and depression. Also, as individuals age, cognitive function plays a key role in predicting physical function and psychological well-being.

